# Influence of "live high-train low" on hemoglobin mass and post-exercise hepcidin response in female endurance athletes

**DOI:** 10.1007/s00421-025-05762-w

**Published:** 2025-04-10

**Authors:** Titta Kuorelahti, Johanna K. Ihalainen, Vesa Linnamo, Claire Badenhorst, Oona Kettunen, Ritva S. Mikkonen

**Affiliations:** 1 Sports Technology Unit Vuokatti, Faculty of Sport and Health Sciences, University of Jyväskylä, Vuokatti, Finland; 2 School of Sport, Exercise and Nutrition, Massey University, Auckland, New Zealand

**Keywords:** Hepcidin, Hemoglobin mass, Normobaric hypoxia, Iron status, Inflammation

## Abstract

**Purpose:**

The aim of this study was to investigate the effects of a 21-day ‘live high-train low’ (LHTL) intervention on hemoglobin mass (Hb_mass_) and post-exercise hepcidin response in female endurance athletes.

**Methods:**

15 national to international level female endurance athletes completed either the LHTL intervention in normobaric hypoxia (2500 m, ~ 18 h·day^−1^, INT, n = 7) or lived and trained in normoxia for the same duration (CON, n = 8). Tests were conducted before (PRE) and within two days after (POST) the intervention including Hb_mass_ measurements via a carbon monoxide rebreathing method and a roller skiing skate test. Venous blood samples were collected at rest, 0, and 3 h after the aerobic exercise to test for changes in serum hepcidin, ferritin, and interleukin-6 (IL-6).

**Results:**

Normobaric hypoxia increased Hb_mass_ (3.3 ± 1.8%, p < 0.001) in INT, while no changes were observed in CON. There were no changes in performance parameters, resting levels of hepcidin, or IL-6 from PRE to POST, but ferritin decreased in both groups (p = 0.040). Hepcidin increased 0 h post-exercise in PRE for INT (p = 0.029) and both 0 and 3 h post-exercise for CON (p = 0.001, p = 0.019). In POST elevated post-exercise hepcidin was only observed in CON (0 h, p = 0.003; 3 h, p = 0.008).

**Conclusions:**

21-day LHTL increased Hb_mass_ and suppressed post-exercise hepcidin response after intensive aerobic exercise. This suggests that prolonged hypoxia may induce an acute physiological response that supports iron absorption within a few days following hypoxic exposure, which may assist in achieving the aerobic adaptations sought from prolonged hypoxic training camps.

## Introduction

Altitude training is a commonly used method among endurance athletes to improve aerobic capacity. The method is mainly based on the hypoxia-induced changes in erythropoietic activity resulting in accelerated red blood cell maturation (Foley 2008) and increased hemoglobin mass (Hb_mass_) (Saunders et al. 2013) although several non-hematological adaptations also exist (e.g., improved muscle buffering capacity, exercise economy) (Gore et al. 2007). The increase in Hb_mass_ may contribute to improved oxygen carrying capacity and maximal oxygen uptake (VO_2max_) (Saunders et al. 2013; Schmidt and Prommer 2010) which is a key determinant of endurance performance (Midgley et al. 2007).

To provide Hb_mass_ and sea level performance adaptations, a sufficient hypoxic dose is required (Gore et al. 2013; Nummela et al. 2021). The current recommendations for altitude training camps involve living at moderate altitude (> 2000 m) for 3–4 weeks (Lundby et al. 2012) where the change in Hb_mass_ occurs with an approximate rate of 1.1% / 100 h of exposure (Gore et al. 2013). As long as the total hypoxic dose of exposure remains sufficient (time spent at altitude, severity of altitude), physiological responses can be attained by using different forms of altitude training, including the traditional live high-train high (LHTH) and contemporary live high-train low (LHTL) performed either in natural altitude or in normobaric hypoxia (e.g. hypoxic apartments) (Garvican-Lewis et al. 2016).

In addition to the hypoxic dose, iron availability has been proposed to influence Hb_mass_ adaptations among athletes (Stellingwerff et al. 2019). Adequate iron supply is important as the transition to altitude places a three- to five-fold increase in erythropoietic iron demand (Reynafarje et al. 1959). Thus, sufficient pre-altitude iron stores (serum ferritin > 30–50 μg·L^−1^) (Stellingwerff et al. 2019; Clénin et al. 2015), as well as iron supplementation before and during altitude exposures, are often recommended to ensure adequate iron bioavailability (Stellingwerff et al. 2019). Of note, the hypoxia-induced accelerated erythropoiesis increases iron availability by indirectly downregulating serum hepcidin concentration via erythroferrone (ERFE) (Kautz et al. 2014). Hepcidin is a small peptide protein mainly responsible for the release and uptake of iron in tissues, as it controls the export to circulation (Agarwal and Yee 2019). Hepcidin acts by binding to the iron-exporting transmembrane protein ferroportin and leads to its degradation, thereby inhibiting iron efflux from the digestive system and iron-recycling macrophages (Nemeth et al. 2004a). The hepcidin response to erythropoietic stimuli occurs relatively quickly, within 1–2 days (Govus et al. 2017). However, the effect of prolonged moderate hypoxia on hepcidin, in turn, remains unclear as both reduced (Govus et al. 2017) and unchanged (McKay et al. 2024; Garvican-Lewis et al. 2018) values have been observed. In addition to the suppression of hepcidin, iron availability in hypoxic environments is supported via gene expression of hypoxia-inducible factors (HIFs) and stimulated transcription of proteins controlling iron absorption (e.g., ferroportin) and transportation (e.g., transferrin) (Gassmann and Muckenthaler 2015).

In contrast to hypoxia and low iron availability, training and exercise have inverse effects on iron regulation. Hepcidin release is up-regulated via exercise-induced inflammatory responses (Peeling et al. 2014; Badenhorst et al. 2014) and predominantly by the increase in inflammatory cytokine interleukin-6 (IL-6) (Nemeth et al. 2004b). The release of hepcidin peaks after exercise with a three-hour delay (Newlin et al. 2012), causing a temporary period of impaired iron absorption and utilization (Barney et al. 2022). Currently, the impact of prolonged hypoxia on post-exercise hepcidin is unclear. Hypothetically, a hypoxia-induced increase in iron demand and lower hepcidin levels at rest might suppress post-exercise hepcidin levels thereby mitigating the disruption to iron absorption post-exercise. Studies have reported conflicting results with a lower post-exercise hepcidin response after a one-week sojourn in moderate altitude (1850 m) (McKay et al 2024), and unaffected hepcidin response after 2-weeks of exposure to normobaric hypoxia (3000 m) (Govus et al. 2017). The exercise-induced changes in IL-6 after prolonged hypoxia also remain unclear: both higher (Mazzeo et al. 2001) and unchanged (McKay et al. 2024) IL-6 responses to endurance exercise have been observed. To the best of our knowledge, this IL-6 response has been measured concurrently with serum hepcidin only in one study (McKay et al. 2024).

Due to the essential role of iron in supporting erythropoietic actions, sufficient iron availability is crucial to ensure the effectiveness of altitude training. For athletes, the role of training and exercise should also be considered as the exercise-induced changes in hepcidin, and inflammation status might limit post-exercise iron availability and therefore influence Hb_mass_ adaptations at hypoxia. These changes in iron regulation are especially interesting in female athletes, who are more prone to iron deficiency than their male counterparts (Clénin et al. 2015), thereby making the role of sufficient iron availability in hypoxia even more significant. As such, this study aimed to investigate the effects of 21-day LHTL on Hb_mass_ as well as post-exercise hepcidin and inflammation responses in female endurance athletes.

## Methods

### Participants

Twenty-one national to international level (Tier 3 to 4) (McKay et al. 2021) female cross-country skiers (17), biathletes (2), and ski-orienteers (2) participated in this study, with each participant self-selecting into either intervention (INT, n = 10) or control groups (CON, n = 11). During the intervention, 5 participants (n = 3 in INT, n = 2 in CON) were excluded from the study due to signs of infection or inability to participate in POST. In addition, one participant (1 × CON) with baseline ferritin < 20 μg·L^−1^ implying iron deficient erythropoiesis (Peeling et al. 2007) was excluded from the analysis. Characteristics of the remaining 15 participants are presented in Table 1. Participants were fully informed of the study procedures and provided written consent to participate. The ethical committee of the University of Jyväskylä approved the study (29/13.00.04.00/2021, January 21, 2021) and all the measurements were conducted in accordance with the Declaration of Helsinki except for pre-registration in a database.

**Table 1:** Characteristics of the participants

	INT *(n* = *7)*	CON *(n* = *8)*
Age (yr)	22 ± 1	23 ± 5
Height (cm)	168 ± 4	165 ± 3
Body mass (kg)	63.1 ± 4.2	59.4 ± 4.1
Fat %	15.8 ± 3.6	16.8 ± 5.2
Training background (h·yr^−1^)	690 ± 100	690 ± 80

### Experimental design

In this longitudinal study, INT performed a 21-day LHTL intervention, while CON lived and trained in normoxia for the same duration. All the measurements were performed during the athletes’ preparatory season in late July—mid-September. INT lived in hypoxic apartments of the Olympic Training Centre (Vuokatti Sport, ∼ 150 m above sea level) with ambient air set to simulate 2500 m (fraction of inspired oxygen ~ 15.8%). The normobaric hypoxic conditions were monitored with Dräger Pac8500 (Dräger, Germany). The participants spent approximately 18 ± 1.6 h·day^−1^ in hypoxia with the corresponding hypoxic dose being 940 km·h (Garvican-Lewis et al. 2016).

The performance and Hb_mass_ measurements were performed on two separate days one to two days before and after the 21-day intervention. All tests were conducted at the same time of day to avoid the influence of circadian variation. On performance test day, all participants first arrived at the laboratory between 7–8 am for fasted venous blood samples and anthropometric measurements. Height was measured with a stadiometer while body composition and mass were measured by using bioimpedance (Inbody 770, Biospace Co., Seoul, Korea). After the morning measurements, the participants were allowed to leave the laboratory and have breakfast before coming back for the performance tests. To avoid variation in nutritional status, participants recorded a food diary 36 h before the performance test in PRE (previous day and the test morning) and then replicated the recorded diet in POST. In addition, the participants were instructed to refrain from caffeine, alcohol, nicotine, and antihistamines for 24 h and exercise for 12 h before arriving for morning blood samples. After the performance test, venous blood samples were collected at the immediate conclusion of exercise and after 3 h of post-exercise recovery to identify the responses in IL-6 and hepcidin. All participants consumed a recovery drink after the first post-exercise blood sample which included 1.2 g·kg^−1^ carbohydrate (Malto Energy, SportLife Nutrition), 0.3 g·kg^−1^ whey protein (Whey protein, SportLife Foods), and 400 ml of water.

Iron supplementation was monitored but not controlled during the intervention. Four participants in INT consumed iron supplements as recommended by their doctors or coaches, with the approximate elemental intake being 31 ± 51 mg·day^−1^ (20–200 mg·day^−1^). The participants were instructed to maintain the same three-week supplementation regimen both 3 weeks before PRE and during the three-week intervention. To avoid the acute effect of supplemental iron on serum iron markers (Moretti et al. 2015), the participants were instructed not to supplement one week prior to both PRE and POST. In CON, none of the participants consumed iron supplements.

### Aerobic exercise test

The physical exercise test was performed by roller ski skating on a treadmill (RL 3500E, Rodby, Södertalje, Sweden). The participants were already accustomed to treadmill skiing or were provided a one-hour familiarization trial prior to PRE. All participants used the same roller skis (Marwe Skating 610, Marwe Oy, Hyvinkää, Finland). The exercise lasted altogether 60 min and included a 20-min warm-up, an incremental exercise test (IXT) until voluntary exhaustion, and a cool-down. The inclination of the treadmill remained constant (3°) throughout the exercise. The velocity of the treadmill was 8–9.5 km·h^−1^ for warm-up and cool-down while during the IXT it increased by 1.5 km·h^−1^ every three minutes. Time to exhaustion (TTE) in IXT was used to describe endurance performance. The cool-down started immediately after the cessation of IXT and lasted until the 60-min total was reached. Respiratory gases were measured continuously throughout the IXT with a mixing chamber system (Cosmed K5, Rome, Italy). Volume and gas calibrations were completed before each measurement. The VO_2max_ was calculated as the highest 60 s average.

### Venous blood samples

Venous blood samples were collected from an antecubital vein and drawn into two 6 ml Vacuette serum clot activator tubes and a 4 ml EDTA gel serum tube (Greiner-Bio-One GmbH). Immediately after the collection, the EDTA tubes were refrigerated and later transported to Vita laboratory (Helsinki, Finland) for basic blood count analysis. The samples were analyzed within 36 h with Sysmex XN-1000 (SysmexCo.).

The serum samples were allowed to clot for 30 min after which they were centrifuged at 1825×*g* for 10 min. The serum was divided into aliquots and stored first at − 20 and then at − 80 until further analysis. For the analysis of circulating hepcidin, enzyme-linked immunosorbent assay with commercial reagents was used (Quantikine human hepcidin ELISA, R&D Systems Inc., Minneapolis, MN, USA). IL-6, high-sensitivity C-reactive protein (hs-CRP), and serum ferritin were analyzed using the Immulite 2000 and immunoassay kits (Immulite, Siemens, IL, USA). The detection limits and inter-assay coefficients of variation were 3.8 pg·mL^−1^ and 8.0% for hepcidin, 2.0 pg·mL^−1^ and 5.3% for IL-6, 0.1 mg·L^−1^ and 8.3% for hs-CRP, and 0.4 μg·L^−1^ and 12.1% for serum ferritin.

### Hemoglobin mass measurement

Hb_mass_ and plasma volume were calculated using the optimized carbon monoxide (CO) rebreathing method (Schmidt and Prommer 2005) and SpiCo Calculation Software 2.2 (Blood tec GmbH, Bayreuth, Germany). In the measurement, the participants inspired the O_2_–CO gas mixture (O_2_ ∼3L; CO 0.9 ml·kg^−1^) for two minutes via a closed-circuit spirometer (Blood tec GmbH, Bayreuth, Germany) in a seated position. Capillary blood samples were collected three times before and twice at 6 and 8 min after the rebreathing procedure, to analyze the fraction of carboxyhemoglobin (%HbCO) with an ABL90 FLEX blood gas analyzer (Radiometer Medical ApS, Brønshøj, Denmark). The changes in %HbCO were used to calculate the Hb_mass_. A typical error reported for the method is 1.7% (Schmidt and Prommer 2005). For plasma volume calculations, hemoglobin concentration and hematocrit analyses from venous blood samples collected before the rebreathing procedure were utilized.

### Training monitoring

During the intervention, participants' training volume and intensity distribution were monitored, and the data was stored online with Polar GPS-enabled watches (Polar Flow, Polar Electro Oy, Kempele, Finland). The monitoring started three weeks before PRE and continued until POST. The training was divided into four categories based on the participants’ heart rate distribution during training: low-intensity training (LIT, target blood lactate < 2.5 mmol·L^−1^), moderate-intensity training (MIT, target blood lactate 2.5–4.0 mmol·L^−1^), high-intensity training (HIT, target blood lactate 4.0–10.0 mmol·L^−1^) (Sandbakk and Holmberg 2017), and strength training (ST).

### Statistical analyses

Results are presented as mean and standard deviation (± SD). The statistical analysis was performed using SPSS Statistics 28 (IMB). The assumption of normality was assessed with the Shapiro–Wilk test and non-normally distributed data was log-transformed (IL-6, hs-CRP). Later, the transformed values were back transformed to their original scale for the figures. A series of mixed ANOVAs with experiment (PRE vs. POST) and time (Baseline, 0 h, and 3 h post-exercise) as within-subject factors and group (INT vs. CON) as a between-subject factor was used to analyze changes in performance, Hb_mass_, and circulating blood parameters. In POST, circulating blood parameters were adjusted for changes in plasma volume (Shrek et al. 2013). If the sphericity assumption was violated, the Greenhouse–Geisser correction was applied. In case of significant interaction, pairwise comparisons with Bonferroni correction were analyzed. Mixed ANOVA was also used to investigate whether there were changes in three-week training volume, intensity distribution, or 36 h energy and carbohydrate intake prior to PRE and POST. In case of significant effect, the change within the variable was used as a covariate to detect whether it influenced Hb_mass_ or hepcidin responses. Cohen’s d effect sizes were calculated and interpreted in accordance with previous literature as follows: d = 0.2–0.6 (small), d = 0.6–1.2 (moderate), and d > 1.2–2.0 (large) (Govus et al. 2017). Significance was accepted as the alpha level of p < 0.05.

## Results

### Maximal performance measures in aerobic exercise test

Maximal performance measures in aerobic exercise tests in PRE and POST are presented in Table 2. No changes or between-group differences were observed in TTE or VO_2max_ regardless of the group.

**Table 2:** Maximal performance measures from incremental exercise test before (PRE) and after (POST) 21 days of LHTL (INT) and living and training in normoxia (CON)

									Mixed ANOVA			
	INT				CON				Main effects			Interaction effects
	PRE	POST	Effect size		PRE	POST	Effect size		group	experiment		group x experiment
TTE (min)	23.6 ± 2.7	23.4 ± 2.4	**0.07**		22.6 ± 1.7	22.6 ± 1.9	**− 0.02**		p = 0.394	p = 0.857		p = 0.802
VO_2max_ (L·min^−1^)	4.0 ± 0.5	3.9 ± 0.4	**0.1**		3.5 ± 0.2	3.7 ± 0.2	**− 0.58**		p = 0.059	p = 0.652		p = 0.344
VO_2max_ (mL·kg^−1^·min^−1^)	62.2 ± 5.5	60.2 ± 4.7	**0.0**		59.4 ± 3.0	60.6 ± 3.5	**− 0.39**		p = 0.266	p = 0.636		p = 0.636

Bold is to highlight the effect sizes, distinguishing them from the actual values of the variables measured at PRE and POST

### Hb_mass_ and circulating blood parameters

**Fig. 1:**
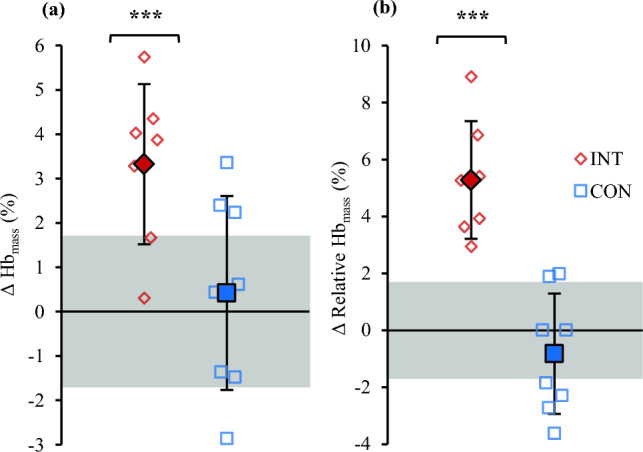
Changes in Hb_mass_ (**a**) and Hb_mass_ relative to body mass (**b**) following 21 days of LHTL (INT) and living and training in normoxia (CON). Diamonds represent INT and squares CON. Grey box describes the typical error of the carbon monoxide rebreathing method. Significant change ***p < 0.001

Overall means in Hb_mass_, plasma volume, and circulating blood parameters in PRE and POST are presented in Table 3. The experiment had a significant main effect on absolute (F = 13.2, p = 0.003) and relative Hb_mass_ (F = 16.6, p = 0.001). In addition, experiment x group interaction was observed in absolute (F = 9.2, p = 0.010) and relative Hb_mass_ (F = 31.8, p < 0.001). As shown in Fig. 1, absolute Hb_mass_ increased by 3.3 ± 1.8% (p < 0.001) and relative Hb_mass_ by 5.3 ± 2.1% (p < 0.001) in INT, while no changes were observed in CON (0.8 ± 2.5%, p = 0.667 / −0.4 ± 2.3%, p = 0.272). There was an experiment x group interaction in plasma volume (F = 8.4, p = 0.013), although post hoc analysis revealed no changes or between-group differences. For relative plasma volume, no main effects or interactions were detected.

**Table 3:** Hemoglobin mass, plasma volume, and resting iron status and iron regulatory markers before (PRE) and after (POST) 21 days of LHTL (INT) and living and training in normoxia (CON)

							Mixed ANOVA		
	INT			CON			Main effects		Interaction effects
	PRE	POST	Effect size	PRE	POST	Effect size	Group	Experiment	Group x experiment
Hb_mass_ (g)	661 ± 63	683 ± 69^aaa^	**− 0.36**	629 ± 58	631 ± 51	**− 0.04**	p = 0.202	p = 0.003**	p = 0.010*
Rel. Hb_mass_ (g·kg^−1^)	10.3 ± 0.6	10.9 ± 0.6^aaa^	**− 1.04**	10.5 ± 0.9	10.5 ± 0.9	**− 0.11**	p = 0.788	p = 0.001**	p < 0.001***
Plasma volume (L)	3.4 ± 0.4	3.2 ± 0.4	**0.49**	3.0 ± 0.4	3.2 ± 0.3	**− 0.56**	p = 0.251	p = 0.936	p = 0.013*
Rel. Plasma volume (mL·kg^−1^)	52.8 ± 5.4	50.8 ± 4.9	**0.42**	50.0 ± 7.6	52.4 ± 6.4	**− 0.37**	p = 0.835	p = 0.859	p = 0.058
Hemoglobin (g·L^−1^)	136 ± 9	141 ± 3	**− 0.81**	139 ± 11	137 ± 7	**0.20**	p = 0.903	p = 0.474	p = 0.150
Hematocrit (%)	43.6 ± 2.3	43.7 ± 1.8	**− 0.08**	43.5 ± 3.8	43.0 ± 2.6	**0.16**	p = 0.767	p = 0.764	p = 0.566
Serum ferritin (µg·L^−1^)	41.4 ± 20.4	32.8 ± 17.6	**0.49**	51.1 ± 31.2	45.1 ± 24.4	**0.23**	p = 0.380	p = 0.040*	p = 0.685
hs-Crp (mg·L^−1^)	0.66 ± 0.72	0.70 ± 0.62	**− 0.23**	0.49 ± 0.19	0.50 ± 0.19	**− 0.11**	p = 0.995	p = 0.528	p = 0.632

Bold is to highlight the effect sizes, distinguishing them from the actual values of the variables measured at PRE and POST

* Significant effect p < 0.05, ** p < 0.001, *** p < 0.001

^aaa^ Significantly different from PREmeasurements p < 0.001

**Fig. 2:**
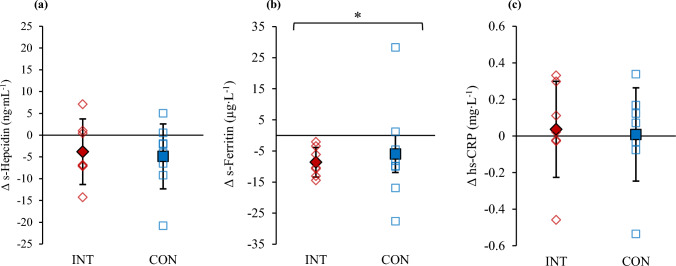
Changes in resting (**a**) serum hepcidin, (**b**) serum ferritin, and (**c**) high-sensitivity C-reactive protein (hs-CPR) following 21 days of LHTL (INT) and living and training in normoxia (CON). Diamonds represent INT and squares CON. Significant effect of time *p < 0.05

**Fig. 3:**
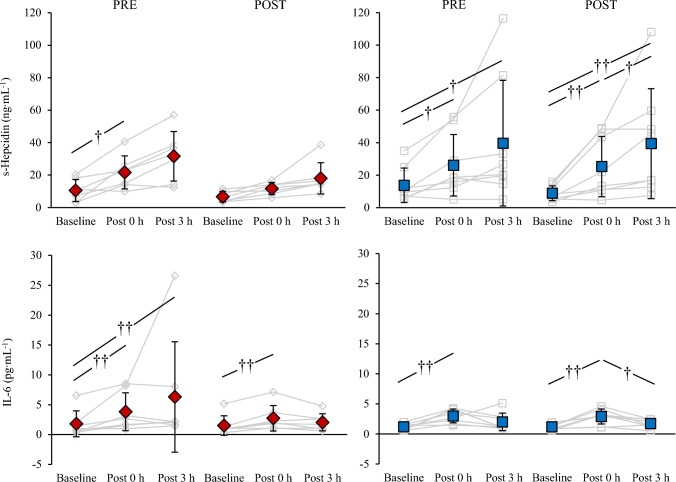
Serum hepcidin and interleukin-6 (IL-6) at baseline, 0 h, and 3 h post-exercise before (PRE) and after (POST) 21 days of LHTL (INT) and living and training in normoxia (CON). Diamonds represent INT and squares CON. Significant difference between time points †p < 0.05, ††p < 0.01

Figure 2 presents changes in resting serum hepcidin, ferritin, and hs-CRP from PRE to POST. There was a significant effect of experiment on resting serum ferritin (F = 5.2, p = 0.040). However, no main group effects or experiment x group interactions were detected, suggesting that the changes were similar across groups. Post hoc analysis was completed to determine specific experiment effects for ferritin, with results indicating no changes or between group differences. For hepcidin no changes or between-group differences occurred regardless of the group (INT p = 0.198, d = 0.77; CON p = 0.086, d = 0.64). In addition, no changes were observed in hs-CRP, resting IL-6 (Fig. 3: INT p = 0.881, d = 0.04; CON p = 0.951, d = −0.04), hemoglobin concentration, or hematocrit (Table 3).

Figure 3 presents serum hepcidin and IL-6 at baseline and in response to aerobic exercise tests in PRE and POST. Time had a main effect on hepcidin (F = 15.6, p < 0.001), and post hoc analyses revealed that hepcidin levels increased in response to exercise among INT from baseline to post 0 h (p = 0.029, d = − 1.39) while the change from baseline to post 3 h was trivial (p = 0.084, d = − 1.93). In POST no changes were observed from baseline to post 0 h or 3 h for INT (p = 0.752, d = − 1.54; p = 0.664, d = − 1.70). In CON hepcidin increased from baseline to post 0 h and 3 h both in PRE (p = 0.01, d = − 0.86; p = 0.019, d = − 0.98) and POST (p = 0.003, d = − 1.30; p = 0.008, d = − 1.35). There was also an effect of time for IL-6 (F = 22.1, p < 0.001), and elevated values for both groups were observed from baseline to post 0 h in PRE (INT: p = 0.008, d = − 1.12; CON: p = 0.004, d = − 1.01) and POST (INT: p = 0.009, d = − 2.25; CON: p = 0.001, d = − 2.92). No main effects of group or experiment on hepcidin (p = 0.254; p = 0.140) and IL-6 (p = 0.705; p = 0.225) were observed, as well as no interaction effects for time x group, time x experiment, group x experiment, or time x group x experiment (hepcidin: p = 0.332, p = 0.812, p = 0.331, p = 0.203; IL-6: p = 0.214, p = 0.057, p = 0.331, p = 0.126).

### Training and nutrition

The mean training volume was 17.8 ± 2.0 h·week^−1^ (LIT 84.8%, MIT 6.1 ± 2.1%, HIT 1.2 ± 1.4%, ST 7.9 ± 2.8%) in INT and 16.3 ± 2.2 h·week^−1^ (LIT 82.5 ± 5.2%, MIT 7.2 ± 3.3%, HIT 1.3 ± 0.7, ST 9.0 ± 3.6%) in CON. No changes or between-group differences were observed in the 3-week training volume prior to PRE and during the 21-day intervention, or in the proportion of MIT, HIT, and ST. In CON there was a decrease in the proportion of LIT (85.4 ± 6.2% to 79.8 ± 5.5%, F = 14.7, p = 0.002) from PRE to POST while no changes were observed in INT. However, the change in LIT did not influence changes in Hb_mass_ or serum hepcidin. No changes or between-group differences were observed in 36 h energy and carbohydrate intake before PRE and POST.

## Discussion

The present study aimed to investigate the effects of 21-day LHTL on Hb_mass_, as well as resting and post-exercise hepcidin and inflammation responses in national to international level (Tier 3 and 4) female endurance athletes. The main finding of the study demonstrated that 21-day LHTL suppressed the post-exercise hepcidin response while no changes occurred in resting or post-exercise IL-6 levels. In addition, 21-day LHTL led to a 3.3% increase in Hb_mass_ in INT although the changes did not contribute to improved aerobic capacity or performance within two days after hypoxic exposure.

### Hb_mass_ and resting blood biomarkers

21-day LHTL (18 h·day^−1^, 940 km·h) produced a 3.3 ± 1.8% (p < 0.001) increase in Hb_mass_ in INT, while no changes occurred in CON. The change in Hb_mass_ was consistent with changes observed in previous studies using a similar hypoxic dose (Garvican-Lewis et al. 2018; Koivisto‐Mørk et al. 2021) as well as with the exponential model estimate presented by Garcivan–Lewis et al. (2016). Although the observed change in Hb_mass_ was significant, there was a relatively high variability in the response among participants and, in fact, two participants in INT were unable to improve their total Hb_mass_ beyond the detection limit of the CO rebreathing method. Hb_mass_ response to altitude training has relatively high variability (Hauser et al. 2017) and can be attributed by many factors such as hypoxic dose, inflammation (Wachsmuth et al. 2013), training (Garvican et al. 2010), energy availability, and iron availability (Stellingwerff et al. 2019). In the present study, no differences in inflammation, training volume, or intensity distribution were observed in INT and therefore these factors were unlikely to have influenced the observed adaptations. The impact of energy availability on Hb_mass_ responses in the current study will be assessed in our upcoming article by Kettunen et al., currently under review.

The effect of iron availability on Hb_mass_ adaptations is critical because supporting increased red blood cell production necessitates a substantial increase in iron demand (Kautz et al. 2014). The present study investigated changes in circulating iron markers serum ferritin and serum hepcidin, both of which tend to respond to hypoxia-induced changes in iron requirements (Garvican-Lewis et al. 2018; Govus et al. 2017; McKay et al. 2024; Garvican et al. 2012). In the present study, only resting ferritin levels were observed to decrease similarly across groups from PRE to POST and no changes were detected in resting hepcidin. The reductions in serum ferritin, however, were small (INT, d = 0.49; CON, d = 0.23) and post hoc, the within group analysis showed no changes from PRE to POST. In the absence of differences in iron intake and exercise levels across groups, the small reduction in ferritin levels following LHTL may suggest increased iron mobilization and utilization for erythropoietic activity. However, as POST was conducted one to two days after the hypoxic exposure, the insights on the effects of changed serum ferritin levels occurring during prolonged hypoxia are limited.

Previously, studies have noted that resting basal iron levels, i.e. ferritin, are prominent determinants of hepcidin expression at rest and in response to exercise (Peeling et al. 2014). As the changes in serum ferritin were small in both groups, only a minimal change in baseline hepcidin was expected. The unchanged resting hepcidin despite the small reductions in serum ferritin may be a result of adequate iron availability and maintenance of iron requirements through dietary intake and supplements during training and hypoxic exposure. This result further supports the current practical recommendation of iron supplement use and dietary iron intake for female athletes to help to maintain iron availability when exposed to prolonged hypoxia (Stellingwerff et al. 2019).

In previous research the hepcidin response to prolonged hypoxia has shown relatively high variability in athletes, as both decreased (Govus et al. 2017) and unchanged (Garvican-Lewis et al. 2018; McKay et al. 2024) values have been reported. The decrease in hepcidin levels in hypoxia occurs mainly due to accelerated kidney erythropoietin (EPO) synthesis, which subsequently increases ERFE production, which in turn, suppresses hepcidin expression (Kautz et al 2014). In sustained hypoxia, EPO levels have been observed to peak within two days, after which the levels tend to decline to baseline over the course of 12 days (Garvican et al. 2012). As the rapid increase in EPO levels in hypoxia appears to result in decreased hepcidin expression within a similar two-day timescale (Govus et al. 2017), in sustained hypoxia the decrease in EPO may also diminish the suppression of hepcidin. Indeed, McKay et al. (2024) found a 35% decrease in hepcidin after 7 days in hypoxia (p < 0.001), whereas, after a 21-day intervention, no changes compared to baseline levels were observed. As such, during prolonged hypoxia of more than two weeks, intra- and extracellular iron concentrations, the other main regulator of hepcidin (Agarwal and Yee 2019), may play a greater role in determining resting hepcidin levels. In the present study, hepcidin was only measured before and one-to-two days after the 21-intervention and thus, the progression of hepcidin levels under hypoxic conditions and during the initial days after returning to sea level cannot be determined. Future studies should monitor how hepcidin, along with ERFE, EPO, and iron status evolve during and after prolonged hypoxic exposures to better understand the changes in iron bioavailability in athletes during hypoxic training camps.

### Post-exercise hepcidin response

Hepcidin levels in the present study increased following aerobic exercise in PRE for both INT and CON. In contrast, after the 21-day intervention, only CON showed a significant increase in post-exercise hepcidin whereas no changes were observed in INT. The suppression in hepcidin response is especially interesting as no differences were found in circulating IL-6 response, one of the primary regulators of serum hepcidin (Nemeth et al. 2004b). Instead, regardless of the group, IL-6 increased significantly after exercise in PRE and POST. The increase in IL-6 in the present study supports the findings of previous research showing an exponential increase in IL-6 following high-intensity exercise, with the highest levels recorded immediately post-exercise (Badenhorst et al. 2014; Peeling et al. 2014). As the IL-6 response and exercise stimulus remained consistent in the present study, we propose that the downregulation of hepcidin response in INT may be due to the previous hypoxic stimulus, which overrode the hepcidin stimulatory effect of exercise-induced inflammation. The present finding is in line with McKay et al. (2014), who found significantly lower hepcidin levels after prolonged low-intensity exercise (60 min at 65% VO_2max_) after a 1-week sojourn in 1800 m, while no changes in IL-6 response were observed. In contrast, Govus et al. (2017) reported conflicting observations detecting no changes in hepcidin response to interval exercise conducted in normoxia and hypoxia after 11 and 14 days of LHTL (⁓14 h·d^−1^, 3000 m). As the studies differ with hypoxic dose, exercise protocols, and participant iron statuses, it is yet to be determined whether there is an optimal recommendation that would support hypoxia-induced changes in post-exercise iron regulation. Nonetheless, the present findings support the utilization of prolonged hypoxia for the short-term suppression of exercise-induced hepcidin production thereby possibly increasing the post-exercise iron export to circulation (Barney et al. 2022). The finding is especially important for athletes prone to exercise-induced anemia, for whom continuous hypoxia may not only provide improved Hb_mass_ and sea level performance adaptations but could also enable improved iron absorption and utilization during post-exercise periods provided that the iron availability and requirements have rebounded to pre-altitude levels. It is also noteworthy, that in the current study, POST was conducted one-to-two days after returning to sea level, suggesting that the beneficial effects of prolonged hypoxia on post-exercise iron regulation may persist for several days after returning to sea level. As such, future research is required to investigate whether the attenuated post-exercise hepcidin response following prolonged hypoxia would provide improved iron absorption in athletes and to determine the durability of the post-exercise hepcidin suppression following sustained hypoxia.

### Hb_mass_ and performance

In the present study, the improvements in Hb_mass_ did not contribute to changes in VO_2max_ or TTE in INT. In addition, no changes in performance parameters were observed in CON. Although prolonged moderate altitude may be effective for increasing both Hb_mass_ and VO_2max_ (Saunders et al. 2013), the scientific evidence remains debated (Millet and Brocherie 2020; Siebenmann and Dempsey 2020). In addition, correlations between the changes in Hb_mass_ and VO_2max_ appear to be relatively weak. Indeed, Hb_mass_ adaptations in hypoxia only seem to explain one-sixth of the variation in VO_2max_, highlighting the role of other hypoxia-induced adaptations, as well as the individual variability in the hypoxia-induced changes in the Hb_mass_-VO_2max_ -relationship (Saunders et al. 2013). As such, the results of the current study imply that despite the beneficial impact of a 21-day LHTL intervention on Hb_mass_, these changes do not necessarily lead to improvements in VO_2max_ or endurance performance. However, it should be noted that the post-measurements of the present study were conducted within two days after returning to sea level. In a meta-analysis by Bonetti and Hopkins (2009), the authors observed increasing VO_2max_ responses with increased post-exposure time and suggested that a greater benefit may appear in VO_2max_ around 2 weeks after altitude intervention. As such, the performance improvements may have appeared after the measurement period in the current study.

### Limitations

Several factors impact iron regulation and therefore may have influenced the results of the present study. Although no scientifically established recommendations for pre-altitude ferritin exist, baseline levels of 30–50 μg·L ^−1^ have been suggested to ensure optimal Hb_mass_ adaptations (Clénin et al. 2015; Stellingwerff et al. 2019). However, in the present study, only four subjects in INT had baseline ferritin over 30 μg·L^−1^ and two over 50 μg·L^−1^. In addition, there was high variability in the supplementation practices and only four out of seven participants in INT consumed iron supplements as recommended by their own doctors and coaches. Most likely, iron supplements were recommended to support the increase in Hb_mass_, although this cannot be determined with the data collected. The approximate supplemental dose in INT was 31 ± 51 mg·day^−1^ (20–200 mg·day^−1^), while the typical recommendation for iron supplementation in altitude has been 100–200 mg·day^−1^ (Govus et al. 2016; Stellingwerff et al. 2019). Although the variability in supplementation practices and low baseline ferritin values did not appear to limit maintaining iron availability in INT, it is possible that these factors accounted for the interindividual variability in Hb_mass_ adaptations.

Low ferritin levels may have also affected hepcidin responses as Peeling et al. (2014) observed that lower baseline ferritin levels were associated with lower resting hepcidin levels and blunted post-exercise hepcidin response. However, as the changes in baseline ferritin were small and the change was similar across groups, this change in basal iron level was unlikely to have a marked influence on hepcidin response following prolonged hypoxia. In addition, despite the use of a female cohort, the study protocol did not allow standardized scheduling of test sessions around the menstrual cycle. As iron regulation may vary across the menstrual cycle (Peeling and McKay 2023), there may have been alterations in the measured iron variables in the present study.

We also acknowledge limitations in the methodology of the study. Firstly, the study was limited to only measuring TTE for describing endurance performance. Although the test does not resemble competition performance, both TTE and VO_2max_ have been observed to be valid predictors of cross-country skiing performance (Talnes et al. 2021), thus providing a feasible estimate of LHTL-induced changes for this group of athletes. The graded exhaustive exercise test protocol used to analyze the responses in post-exercise circulatory markers may also differ from typical high-intensity exercises performed during hypoxic training. However, the serum hepcidin levels at baseline and 3 h post-exercise at PRE were similar to what has been observed in previous studies using high-intensity interval exercises (85–90% vVO_2max_) (Badenhorst et al. 2014; Govus et al. 2017), suggesting that the exercise protocol used in the present study was appropriate for investigating post-exercise hepcidin responses following prolonged hypoxia.

Finally, limitations existed in the Hb_mass_ assessment. The present study only assessed Hb_mass_ once at PRE and POST instead of using duplicate measurements as recommended for athletes by Hauser et al. (2017). Despite our attempt to mitigate measurement error in the Hb_mass_ assessment through the collection of multiple blood samples from a fingertip, conducting a single rebreathing protocol may have influenced the precision of the measurement and therefore the ability to detect the hypoxia-induced changes in Hb_mass_.

## Conclusions

The results of the present study indicate that prolonged LHTL increases Hb_mass_ and suppresses post-exercise hepcidin response in female endurance athletes one to two days after hypoxic exposure. The reduced response may allow improved iron absorption and recycling post a training stimulus which, in turn, may support accelerated red blood cell production in the acute time frame following prolonged hypoxia. Further research is required to determine the duration of the acute suppression of hepcidin following prolonged hypoxia and whether it is associated with changes in post-hypoxic performance.

## Data Availability

The datasets generated during and/or analyzed during the current study are not publicly available due to reasons of sensitivity but are available from the corresponding author on reasonable request.

## Abbreviations

%HbCO: Fraction of carboxyhemoglobin
CO: Carbon monoxide
CON: Control group
EPO: Erythropoietin
ERFE: Erythroferrone
Hb_mass_: Hemoglobin mass
HIFs: Hypoxia-inducible factors
HIT: High-intensity training
hs-CRP: High-sensitivity C-reactive protein
IL-6: Interleukin-6
INT: Intervention group
IXT: Incremental exercise test
LHTL: Live high-train low
LHTH: Live high-train high
LIT: Low-intensity training
MIT: Moderate-intensity training
TTE: Time to exhaustion
VO_2max_: Maximal oxygen uptake
